# AI-assisted tracking of worldwide non-pharmaceutical interventions for COVID-19

**DOI:** 10.1038/s41597-021-00878-y

**Published:** 2021-03-25

**Authors:** Parthasarathy Suryanarayanan, Ching-Huei Tsou, Ananya Poddar, Diwakar Mahajan, Bharath Dandala, Piyush Madan, Anshul Agrawal, Charles Wachira, Osebe Mogaka Samuel, Osnat Bar-Shira, Clifton Kipchirchir, Sharon Okwako, William Ogallo, Fred Otieno, Timothy Nyota, Fiona Matu, Vesna Resende Barros, Daniel Shats, Oren Kagan, Sekou Remy, Oliver Bent, Pooja Guhan, Shilpa Mahatma, Aisha Walcott-Bryant, Divya Pathak, Michal Rosen-Zvi

**Affiliations:** 1 IBM Research, Yorktown Heights, USA; 2 IBM Research, Cambridge, USA; 3 IBM Research, Nairobi, Kenya; 4 IBM Research, Mount Carmel Haifa, Israel

**Keywords:** Health policy, Health care economics, Databases

## Abstract

The Coronavirus disease 2019 (COVID-19) global pandemic has transformed almost every facet of human society throughout the world. Against an emerging, highly transmissible disease, governments worldwide have implemented non-pharmaceutical interventions (NPIs) to slow the spread of the virus. Examples of such interventions include community actions, such as school closures or restrictions on mass gatherings, individual actions including mask wearing and self-quarantine, and environmental actions such as cleaning public facilities. We present the Worldwide Non-pharmaceutical Interventions Tracker for COVID-19 (WNTRAC), a comprehensive dataset consisting of over 6,000 NPIs implemented worldwide since the start of the pandemic. WNTRAC covers NPIs implemented across 261 countries and territories, and classifies NPIs into a taxonomy of 16 NPI types. NPIs are automatically extracted daily from Wikipedia articles using natural language processing techniques and then manually validated to ensure accuracy and veracity. We hope that the dataset will prove valuable for policymakers, public health leaders, and researchers in modeling and analysis efforts to control the spread of COVID-19.

## Abstract (summary)

**Table:** 

Measurement(s)	Preventive Intervention • Public Health
Technology Type(s)	natural language processing objective • Artificial Intelligence
Sample Characteristic - Environment	anthropogenic environment
Sample Characteristic - Location	Global

Machine-accessible metadata file describing the reported data: 10.6084/m9.figshare.13999484

## Background & Summary

The Coronavirus disease 2019 (COVID-19) pandemic has made an unprecedented impact on almost every facet of human society from healthcare systems to economies and governments worldwide. As of January 2021, almost every country in the world has been affected, with more than 94 million confirmed cases of infection and a worldwide death toll over 2 million cases^1–3^. Most countries have resorted to non-pharmaceutical interventions (NPIs) as a primary strategy^4,5^ for disease control. According to the United States Centers for Disease Control and Prevention (CDC), (NPIs) are “actions, apart from getting vaccinated and taking medicine, that people and communities can take to help slow the spread of illnesses like pandemic flu” (https://www.cdc.gov/nonpharmaceutical-interventions). Examples of such interventions include community actions (e.g., school closures, restrictions on mass gatherings) and individual actions (e.g., mask wearing, self-quarantine). Such NPIs vary significantly in their implementation in different countries based on the maturity of the health infrastructure, robustness of the economy, and cultural values unique to the region.

Public health policy makers worldwide are striving to incorporate successful intervention plans to manage the spread of disease while balancing its socio-economic impact^6,7^. These initiatives can benefit from technologies that model the efficacy of different intervention strategies. The pandemic has sparked an ongoing surge of discovery and information sharing, resulting in an unprecedented amount of data being published online^8^. This includes information about NPIs, which are available in a wide variety of unstructured data sources, including official government websites^9,10^, press releases, social media, and news articles. However, such modeling requires the information about the NPIs to be available in a structured form.

To address this urgent need, a number of data collection initiatives have emerged in recent months resulting in several datasets with varying degrees of coverage, data freshness, and granularity of details. A comparison of datasets can be found in the Supplementary Information file. Most of these efforts, such as the Complexity Science Hub Covid-19 Control Strategies List (CCCSL)^11^ and the Oxford COVID-19 Government Response Tracker (OxCGRT)^12^, use labor-intensive crowdsourcing to curate the datasets. In order to reduce the manual effort, hybrid approaches such as CoronaNet^13^ employ natural language processing (NLP) based automated methods to identify potential NPIs from news articles and manually validate them. However, this results in a substantial redundancy of NPIs within the dataset because these approaches lack the ability to uniquely reference different mentions of an NPI, both within and across articles. As a result, more volunteering effort is required to verify and normalize these repeated NPIs. Furthermore, given that the NPI description is often spread over several sentences in news articles, automated NPI extraction, along with its attributes, requires a higher-level information extraction framework that can resolve the inter-sentential/inter-paragraph relationships expressed in the text. These deficiencies in existing approaches call for a better method to address this critical problem.

**Fig. 1:**
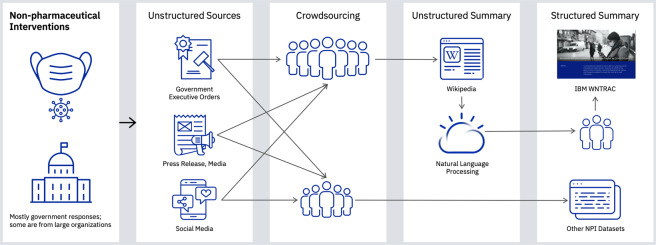
AI-assisted approach to build the Worldwide Non-pharmaceutical Interventions Tracker for COVID-19 (WNTRAC) dataset.

Wikipedia contains a large number of articles on the COVID-19 pandemic; this includes government-implemented NPIs, which are continuously updated. In contrast to the news articles, Wikipedia has the full description of the NPIs written succinctly, often in a single sentence. Moreover, the NPIs related to a specific topic are often organized within the article by location or date. As such, we postulated that an approach, as shown in Fig. 1, based on automated information extraction from Wikipedia, followed by human validation to ensure accuracy and veracity, would result in a high-quality, frequently-updated dataset but with far less manual effort.

We present the result of our work on the Worldwide Non-pharmaceutical Interventions Tracker for COVID-19 (WNTRAC), a comprehensive dataset consisting of over 6,000 NPIs implemented worldwide since the start of the pandemic. WNTRAC covers NPIs implemented across 261 countries and territories, and classifies NPIs into a taxonomy of 16 NPI types. Our novel WNTRAC automated NPI curation system, open-sourced along with the dataset, keeps the dataset up-to-date with just two person-hours of effort of manual validation per day.

In what follows, we explain the methods used to create the dataset, outline the challenges and key design choices, describe the format, and provide an assessment of its quality. We also lay out our vision of how this dataset can be used by policy makers, public health leaders, and data scientists and researchers to support modeling and analysis efforts.

## Methods

Wikipedia is one of the main sources of accessible information on the Internet. Since the start of COVID-19, a dedicated global network of volunteers has been creating, updating, and translating Wikipedia articles with vital information about the pandemic. Nearly 7,000 new Wikipedia pages on COVID-19 have been written by more than 97,000 volunteers (https://wikimediafoundation.org/covid19/data) since the onset of the pandemic, which have accumulated more than 579 million page views by January 2021 (up from 440 million in August 2020 when the preprint of this work was first published). Wikipedia articles are crowd-sourced through the process of collective validation^14^ and by citations of credible sources such as government websites, scientific literature, and news articles; this allows them to serve as a reliable source of NPI data. Furthermore, these Wikipedia articles are constantly being updated. They have been edited more than 917,000 times as of December 2020 (compared to 793,000 in August 2020), making it both a rich and up-to-date source. We built a semi-automated system to construct the dataset from Wikipedia articles and keep it current with their updates.

**Fig. 2:**
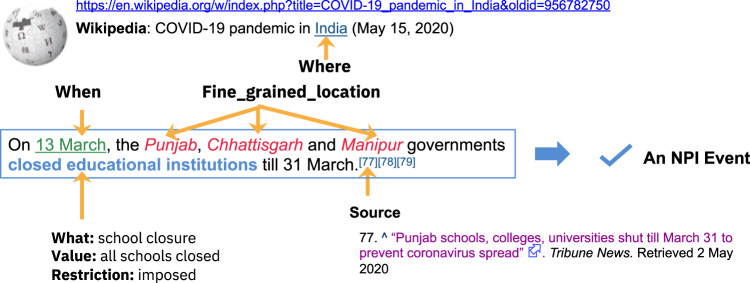
An example of the NPI mentioned in the Wikipedia article of 15^th^ May 2020.

The NPIs are modeled as events and evidences for information extraction purposes. This is illustrated by a motivating example shown in Fig. 2. Each event corresponds to an imposition or lifting of a particular NPI. An event is defined to be a 5-tuple (what, value, where, when, restriction), in which

1. What: the *type* of NPI that was imposed or lifted. NPIs are grouped into 16 major types. In the example, the type is *school closure*.
2. Value: sub-category or attribute that further qualifies the NPI type more specifically. In the example, the associated value is *all schools closed*. A detailed description of each type and the corresponding possible values is shown in Table 1.

**Table 1:** Taxonomy of the Worldwide Non-pharmaceutical Interventions Tracker for COVID-19 dataset.

NPI	Example	Value	Value description
Prison-related Policies	On March 30, the GNA announced the release of 466 detainees in Tripoli, as part of an effort to stop the spread of the virus in prisons.	Integer	Number of prisoners that were released
Confinement	On 19 March, President Alberto Fernández announced a mandatory lockdown to curb the spread of coronavirus.	Category	1. Mandatory/advised for all the population. 2. Mandatory/advised for people at risk
Contact Tracing	On 2 March, a case in Nimes was traced to the mid-February Mulhouse Megachurch event.	Category	1. Tracing back 14 days of contacts of a confirmed patient through electronic information. 2. Tracing contacts of a person who needs to be isolated as was in contact with a confirmed patient through electronic information
Domestic Flight Restrictions	On 1 April, the Government of Afghanistan suspended flights between Kabul and Herat.	String	Name of the state where the passenger is arriving from
Economic Impact	Up until 14 March, the Afghan government had spent $25 million to tackle the outbreak, which included $7 million of aid packages.	Category	1. Stock market 2. Unemployment rate 3. Industrial production
Entertainment/Cultural Sector Closures	On April 7, Rockland and Sullivan counties closed their parks.	Category	1. Bars, restaurants, night clubs 2. Museums, theaters, cinema, libraries, festivities 3. Parks and public gardens 4. Gyms and pools 5. Churches
Freedom of Movement (International)	Iran was added to the list of countries whose nationals were suspended entry to Cambodia, making a total of six.	String	Name of the country the citizen is from
International Flight Restrictions	With effect from midnight on 1 April, Cuba suspended the arrival of all international flights.	String	Name of the country or state where the passenger is arriving from
Travel Quarantine Policies	Israeli nationals returning from Egypt were required to enter an immediate 14-day quarantine.	String	Name of the country or state where the passenger travelled from
Mask Wearing	On April 15, Cuomo signed an executive order requiring all New York State residents to wear face masks or coverings in public places.	Category	1. Mandatory 2. Mandatory in some public spaces 3. Recommended
Restrictions on Gatherings	On 13 March, it was announced at an official press conference that a four-week ban on public gatherings of more than 100 persons would be put into effect as of Monday 16 March.	Integer	Maximum number of people in social gatherings allowed by the government
Public Service Closures	On 19 March, Election Commissioner Mahinda Deshapriya revealed that the 2020 Sri Lankan parliamentary election will be postponed indefinitely until further notice due to the coronavirus pandemic.	Category	1. Government/parliament system closed 2. Legal system closed
Public Transportation	On March 20, Regina Transit and Saskatoon Transit suspended fares for all bus service, but with reduced service.	Category	1. Partial cancellation of routes/stops during the week/weekend 2. Total cancellation of transport (special case for some states in China)
School Closures	On 13 March, the Punjab and Chhattisgarh governments declared holidays in all schools and colleges till 31 March.	Category	1. All schools (general) closed 2. Only kindergartens/daycare closed 3. Only schools (primary/secondary) closed 4. Universities closed
State of Emergency	Governor Charlie Baker declared a state of emergency for the state of Massachusetts on March 10.	Category	1. National guard joins the law enforcement 2. Army joins the law enforcement
Work Restrictions	On 10 April, Koike announced closure requests for six categories of businesses in Tokyo.	Category	1. Suggestion to work from home for non-essential workers 2. Mandatory work from home enforcement for nonessential workers
3. Where: the region (country, territory, province, or state) in which the NPI has been implemented or withdrawn. In this example, the associated region will be “India”.
4. When: The date from which the NPI was imposed or lifted. In the example, the date will be *13 March*, corresponding to the implementation of the NPI, even if a likely date for the cancellation of the NPI, *31 March*, is indicated.
5. Restriction: a flag indicating that the event corresponds to the introduction or withdrawal of the NPI. It should be noted that the lifting of the NPI is treated as a separate event. In the example, the restriction type is *imposed*.

In addition to the mandatory fields described above, each event has at least one evidence. An evidence is the sentence text from which the particular event was extracted. In the example, “On 13 March, the Punjab, Chhattisgarh, and Manipur governments declared holidays in all schools and colleges until 31 March” is the evidence. Each evidence is accompanied by a source type indicating the type of source of Wikipedia citation. Furthermore, we also capture fine grained location information associated with an event. In the example shown, there are three distinct locations, namely, *Punjab, Chhattisgarh, Manipur* that are identified as *fine_grained_location*. More details about such additional attributes can be found in the data records section.

**Fig. 3:**
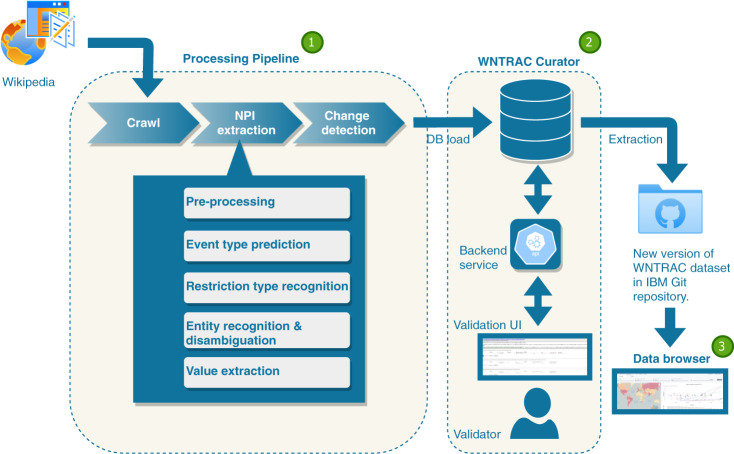
The WNTRAC system consisting of a processing pipeline, WNTRAC Curator validation tool and NPI data browser.

The system, shown in Fig. 3, is designed to be scalable for the continuous gathering, extraction, and validation of NPI events. It consists of three subsystems: a data processing pipeline for capturing and extracting potential NPI events from Wikipedia articles, a tool called WNTRAC Curator for the human validation of NPI events that were automatically extracted using the aforementioned pipeline, and a data browser for visualizing the data. In the next section, we describe the system and its components at a high level. We focus on key design choices that have a bearing on the quality of the dataset, starting with a brief description of the data collection.

### Data collection

As stated earlier, Wikipedia includes a broad range of articles on COVID-19 covering a variety of topics, including the cause, transmission, diagnosis, prevention, management, economic impact, and national responses. Categories are used in Wikipedia to link articles under a common topic and are found at the bottom of the article page. This dataset was collected by automatically crawling Wikipedia articles discussing COVID-19 in different regions, belonging to the category (https://en.wikipedia.org/wiki/Help:Category) COVID-19 pandemic by country. There were 384 articles under the category, COVID-19 pandemic by country when retrieved recursively as of July 2020. We prioritized a set of 261 out of these 384, which includes all the countries that had reported an NPI along with 54 US states and territories. The remaining 123 (384−261) articles recorded NPI events at smaller administrative levels, such as cities or counties. The list of supported regions are available in the Supplementary Information file. For *Mask Wearing* NPI type alone, Wikipedia articles were observed to be incomplete for some regions, so we augmented the dataset with a hand-curated list of NPIs from web sources.

### Processing pipeline

The first step in the data processing is to retrieve the aforementioned list of Wikipedia articles on a periodic basis. The crawler module implements this functionality and uses the MediaWiki API (https://wikimedia.org/api/rest_v1/) to download the articles. As part of this step, we extract the text content of each article, while at the same time preserving all the associated citations. This process produces a document for each article. Each sentence in a document is a candidate for NPI extraction. As of January 2021, the aggregate-crawled data contained over 64,000 sentences, with an average of 246 sentences per document. The second step in the pipeline is the extraction of the candidate NPI events from a document. This step is broken into a sequence of sub-steps described below.

- Pre-processing: As the first step in processing a document, we use sentence boundary detection algorithms from libraries such as spaCy^15^, to identify where sentences begin and end. Although the sentences are used as logical units to extract NPI events, we preserved the order in which they appear in the source document for the reasons detailed below. Also, at this step, we extract and retain the citation URL for each sentence, if available.
- Sentence classification: Next, we classify the sentence into one of the NPI types, such as *school closure*, to identify potential NPI events. If no NPI is discussed in the sentence, we classify it as *discarded*. We use multiple learning algorithms, including logistic regression, Support Vector Machines (https://scikit-learn.org/stable/modules/classes.html#module-sklearn.linear_model), and Bidirectional Encoder Representations from Transformers (BERT)^16^, and employ an ensemble method to obtain better overall predictive performance. A small subset of the data (1490 sentences), was manually annotated to train the models. Independently, we also categorize the sentence as implying either the introduction or the withdrawal of an NPI (restriction).
- Named entity recognition and named entity disambiguation: After we identify the potential events in the previous step, we extract specific constituent entities for each candidate event from the sentence. We used state-of-the-art named-entity recognizers (such as spaCy^15^) and normalizers to detect and normalize locations (*Where*: [*Punjab*, *Chattisgarh*, *Manipal*]) and time expressions (*When*: *March 13*). We also link the location entities of type ‘GPE’ in the Wikipedia article title to the corresponding ISO codes. Although we use the sentence as a logical unit for the extraction of an NPI event, the sentence itself may not include all the relevant information. For example, date or location may be available in sentences in the vicinity or in the header of the paragraph to which the sentence belongs. To address this key challenge, we developed a heuristic-based relation detection algorithm to associate one of the extracted dates or locations from the current document to each sentence.
- Value extraction: The last step in NPI event extraction determines the associated value. We use multiple rule-based algorithms that either operate independently or depend on information extracted by the previous steps. For example, given the sentence “On 13 March, it was announced at an official press conference that a four-week ban on public gatherings of more than 100.”, the event type is *mass gathering* and the associated value is *maximum number of people in social-gathering allowed by the government*. The value extraction is performed using parse-based rule engines^15^. It is worth noting that the value extraction components should know the actual type *mass gatherings* before extracting the correct value “100”. Similarly, given a sentence “On 1 April, the Government of USA suspended flights from New York to Texas”, the event type is *domestic flight restriction* and the associated value is *name of the state where the passenger is arriving from*. To correctly extract the value, the value extraction needs to know the correct type and normalized locations (“New York”) respectively.

Thus, using the above procedure, we extract the candidate NPI events (unique 5-tuples) together with their associated evidence (sentences). The next step in the process is change detection. The current version of the Wikipedia article for each region is retrieved during the daily crawl. The article may have been updated since the last time it was fetched, with both new NPI events as well as potential updates to previously recorded events. The goal of this step is to identify these changes in order to prevent duplicate NPI events in the dataset. This is achieved in two ways:

- Remove any candidate NPI events extracted from sentences that have not changed since the last time the Wikipedia article was crawled. This automated elimination of duplicates reduces the validation time since those candidate NPI events are not even shown to the volunteers for validation.
- Flag any possible duplicates, where the wording of the sentence has changed but not the meaning, for further human verification.

To perform this step, we use a combination of syntactic similarity metrics, such as Levenshtein norm, and semantic similarity metrics, such as event attribute matching. This automated change detection greatly reduces the number of duplicates, thus improving the dataset quality while minimizing human labor.

### WNTRAC Curator

**Fig. 4:**
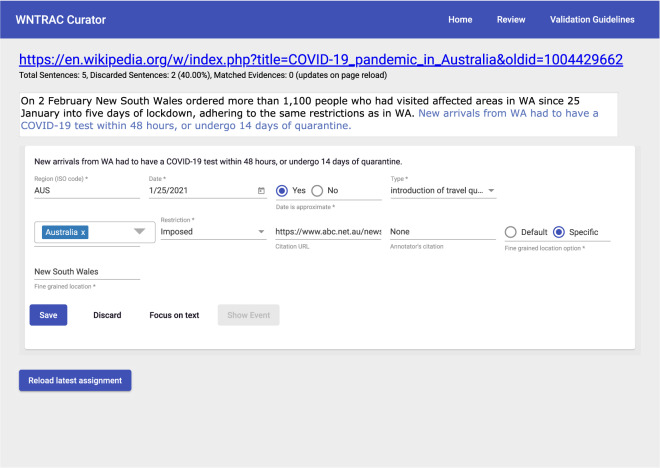
Updated NPI events from a Wikipedia article that are automatically extracted daily by the system are shown to a human volunteer for validation and correction in the WNTRAC Curator tool.

The candidate NPI events from the previous step are presented to the volunteers for validation using the WNTRAC Curator validation tool. The tool is a simple web-application backed by a relational database as shown in Fig. 3. After the change detection, candidate NPI events are loaded into a table in the database. The database also contains previously validated NPI events and their corresponding evidences in separate tables. The tool is shown in Fig. 4. The web-application retrieves all the candidate NPI events pending validation for a region and presents them to a volunteer. At the top, it displays the complete Wikipedia document extracted by the processing pipeline. Below the document, each candidate NPI event, together with the evidence sentence, is shown to the volunteer in separate *cards*. A volunteer performs validation one card at a time. If the card does not contain a valid candidate NPI event, they dismiss it by pressing the Discard button. Otherwise, they start validation by correcting any of the attributes associated with the candidate NPI event extracted by the pipeline. If the sentence does not contain a citation URL, the volunteer might add a URL linking to a source such as a news article or government order that describes the NPI event. After performing any such necessary corrections, the volunteer can persist the updated candidate NPI event by clicking the Save button. Upon saving, if the candidate NPI events does not match any of the previously validated NPI event, it is promoted to become a brand new NPI event and added to the dataset. Otherwise, it is added as a new piece of evidence to an existing NPI event. Clicking the Show Event button, reveals the matched NPI event and its associated evidences. If the newly added evidence is a duplicate of another, the volunteer deletes the previously added evidence. This guarantees that evidences associated with an NPI event are up to date and have no duplication. Thus, the WNTRAC Curator tool adds the human-in-the-loop component to the automated process to ensure data quality. After all the candidate NPI events for a given day are validated, a new version of the dataset can be extracted from the database. All the validated NPI events and their corresponding evidences are extracted into two separate CSV files. The file formats are described in the Data Records section.

### NPI Data Browser

**Fig. 5:**
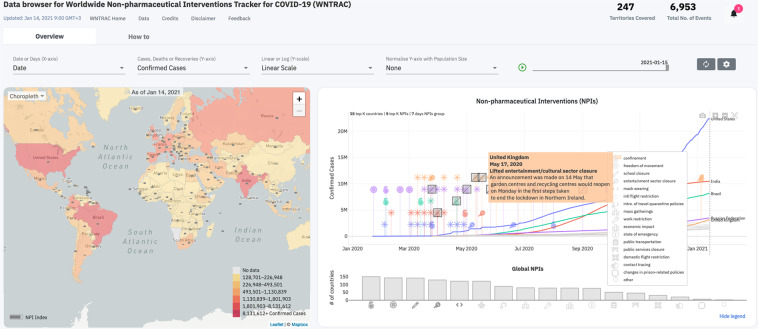
NPI data browser for visualizing the Worldwide Non-pharmaceutical Interventions Tracker for COVID-19 dataset.

Figure 5 presents an interactive data browser (https://covidresponse.res.ibm.com/) that uses a chart, map, and histogram to provide a descriptive analysis of NPIs and COVID-19 outcomes, such as confirmed cases and deaths. The browser has a control panel used to filter the data being visualized (e.g., cases vs. deaths), as well as how it is visualized (e.g., linear vs. log scale). A play slider can be used to view the temporal evolution of NPIs and COVID-19 outcomes in a given region. The chart illustrates the time points at which a geographical region imposes or lifts an NPI along with the temporal trends of COVID-19 outcomes. The different types of NPIs are illustrated using specific icons that are described in a legend. Groups of interventions are noted with a star icon. The number of countries/territories and the number of NPIs shown in the chart can be adjusted in the settings. The user can select a specific line on the chart referring to a territory to focus on the NPIs imposed and lifted in that location. The histogram below the chart shows the number of territories that have imposed the different types of NPIs and can be selected to see the territories on the map that have imposed the selected subset of NPIs. The map illustrates the proportion of NPI categories (out of the 16 NPI categories in the dataset) implemented in each region using a gray-colored bar. Furthermore, when a region is selected, the gray-colored bar in any other region illustrates the proportion of NPI categories in the other region as a proportion of NPI categories implemented in the selected region. The map is also used to visualize the geographic distribution of the selected COVID-19 outcome using choropleth, spikes, or bubbles. The user can interact with the territories on the map to focus on a location and view the data on the chart. Note that for some countries such as the United States, the map can be zoomed to reveal finer-grained data for sub-regions such as states.

## Data Records

Each version of the dataset consists of two CSV files named *ibm-wntrac-yyyy-mm-dd-events.csv* and *ibm-wntrac-yyyy-mm-dd-evidences.csv*, corresponding to events and evidences, respectively. The events are uniquely identified by even_id and evidences by evid_id. All the evidences for a given event have the same even_id. The fields corresponding to the 5-tuple for an event, namely type, country, date, value, and restriction are also present in all its evidences. The evidences record also contains additional fields. These include, citation_url - automatically extracted Wikipedia citation, anno_provided_url - additional citation provided by the volunteer during validation, and fine_grained_location - location entities discussed in the evidence sentence. Table 2 shows a complete listing of fields across event and evidence, along with an example for each of them.

**Table 2:** Data record for the Worldwide Non-pharmaceutical Interventions Tracker for COVID-19 dataset.

Field name	Description	Example
even_id	Globally unique identifier for the particular NPI	7db34fd1-d121-479f-9713-af7596a45aa1
type	Type of the NPI	School closure
country	Country where the NPI was implemented. Name in ISO 3166-1 coding	USA
state/province	State or province where the NPI was implemented. Name in ISO 3166-2 coding	Vermont
date	Date when the npi comes to effect. It is not the date of announcement	2020-03-26
epoch	Unix epoch time corresponding to the date	1589749200000.0
value	Value associated with the npi.	Refer to Table for details
restriction	Ordinal values representing imposition (1) or lifting (0) of an NPI	0
sent_id	Globally unique identifier for the evidence sentence	d68ea644-24d5-4abf-93b0-dabc1cd3c2eb
doc_url	Document URL	https://en.wikipedia.org/wiki/COVID-19_pandemic_in_Vermont
crawl_id	Globally unique identifier for the particular crawl in which this evidence sentence was fetched	2020-05-06_d0cba9ae-8fda-11ea-b351-069b8ffc8dc8
crawl_date	Date of the crawl that fetched this evidence sentence	20200506
text	Evidence sentence in the document where the NPI is discussed	On March 26, Governor Scott ordered all schools in Vermont to remain closed for in-person classes for the rest of the academic year
citation_url	URL cited for the evidence sentence in the source document. In case of multiple, separated by pipeline.	
anno_provided_url	Additional citation URL provided by the human volunteer who performed the validation.	
fine_grained_location	Geographic locations mentioned in the evidence sentence separated by pipeline.	Vermont
source_type	Wikipedia citation source type indicating government (*G*) or other sources (*O*)	G

A live version of the dataset is available in our GitHub repository https://github.com/IBM/wntrac/tree/master/data for download. The dataset is regularly updated. At the time of submission, the dataset was updated as of January 7th, 2021. Historical versions of the dataset are made available in the same GitHub repository. A static copy of the dataset containing NPIs recorded as of 8th July 2020, used for the technical validation in the paper has been archived in figshare^17^. In the next section, we include some high-level dataset statistics to provide a sense of the distribution of the data.

### Dataset statistics

**Fig. 6:**
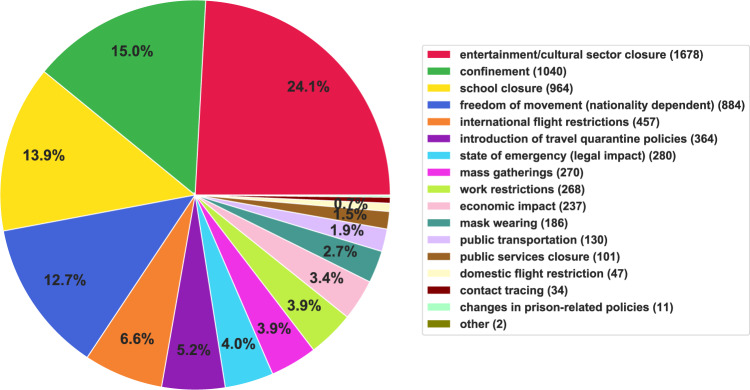
Distribution of NPIs in the Worldwide Non-pharmaceutical Interventions Tracker for COVID-19 dataset.

**Fig. 7:**
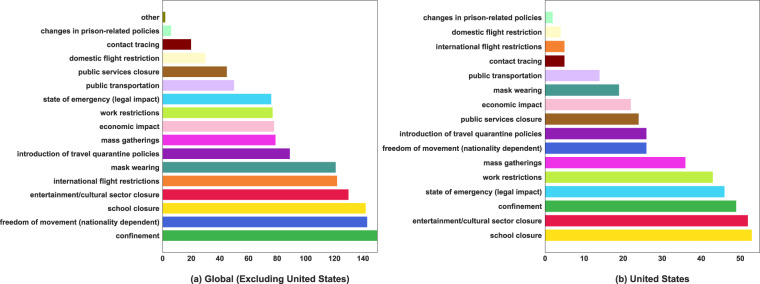
Number of regions implementing each NPI globally and within the United States.

**Fig. 8:**
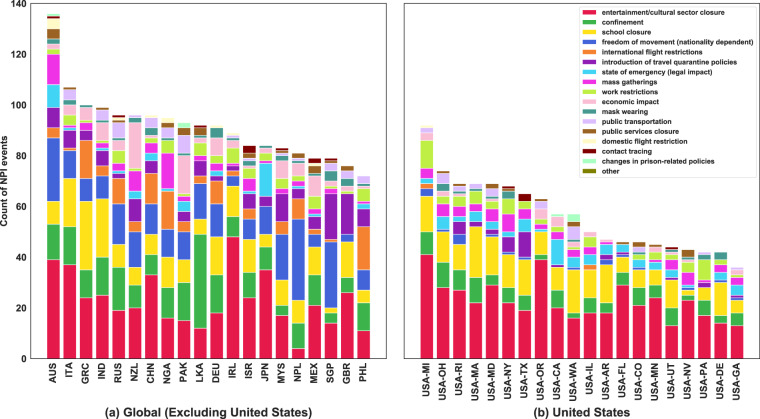
Distribution of NPI implemented in different geographies globally and within the United States.

Figure 6 shows the distribution of the NPIs imposed worldwide as recorded in the dataset. *Entertainment/cultural sector closure*, *confinement* and *school closure* are the predominant NPIs taken by governments. Figure 7 summarizes the total number of regions that implemented NPIs of each type. As shown in the graph, *confinement*, *school closure*, and *freedom of movement* are the most common NPIs imposed worldwide, as expected from Fig. 6. Figure 8 shows the breakdown of the NPIs within each region, for the top 20 regions that implemented the highest number of NPIs. Figures in this section and Usage Notes section were generated from the version of dataset, dated 7th January 2021. A copy of this version of the dataset is also available in figshare^17^. These figures can be generated with any version of the dataset using the Jupyter notebook available at https://github.com/IBM/wntrac/blob/master/code/analysis/dataset_statistics.ipynb.

## Technical Validation

The validation team consisted of a mix of experts who participated in the design of the taxonomy and/or the pipeline, along with IBM volunteers who completed a brief training session about the annotation schema and tool. The validation was done in two stages. In the first phase, because the WNTRAC tool was still being developed, we used simple CSV files to distribute the data for validation. Each annotator was given a complete document corresponding to a Wikipedia article for a particular region, retrieved as on June 6, 2020, pre-annotated with the output of the pipeline. Each sentence was displayed in a separate line, with sentences corresponding to candidate events highlighted with a different background color. The attributes extracted by the pipeline were listed next to each sentence. The annotators were asked to verify and correct each of these attributes. If a sentence did not discuss any of the valid event types, they were asked to mark the type as *discarded*. If a sentence was incorrectly discarded by the pipeline, they were asked to correct the type and fill in the attributes when possible. This was, however, not uniformly enforced. In the second phase, we made the WNTRAC Curator tool available to the annotators. The tool randomly assigns a single document for validation to each annotator. Each document, consists of incremental changes done to the underlying Wikipedia article since the last validation of the document. The validation process for the second phase is similar to the first phase, except that only candidate events, as determined by the pipeline, were shown to the annotators. This time-saving move was based on the observation made during the first phase. When all sentences were presented, human annotators generally agreed with the automated pipeline regarding discarded sentences. The NLP model used a recall-oriented threshold and only discarded sentences with low scores on all valid NPI types.

To determine the quality of the dataset post validation, inter-annotator agreement (IAA) using Cohen’s kappa was calculated on a subset, randomly sampled (2%), from the full set that was validated by IBM volunteers. Each instance in the subset was further double-annotated independently by two experts who were randomly selected from a pool of six experts; this resulted in three sets of annotations per instance. The IAA was evaluated on all five fields of the 5-tuple that uniquely defines an event. Furthermore, the evaluation was performed at a field level for all fields except the value, which is technically a sub-field of type; it does not make sense for it to be analyzed on its own. The IAA results are shown in Table 3. Note that the IAA between experts were consistently high in all categories, indicating that the annotation schema is not ambiguous and most sentences can be consistently assigned to an NPI type defined in the taxonomy. The IAA between the volunteers and experts was also good (0.58) at the NPI type level and the agreement was high (0.81) in the five most frequent NPI types.

**Table 3:** Inter-annotator agreement (measured using Cohen’s kappa) between average volunteers (A) and two groups of experienced volunteers (E_1_ and E_2_). Region includes both country and state/territories as applicable.

	All NPI event types			Top 5 NPI event types		
	A vs E_1_	A vs E_2_	E_1_ vs E_2_	A vs E_1_	A vs E_2_	E_1_ vs E_2_
Type	0.63	0.69	0.80	0.81	0.77	0.85
Type + Value	0.41	0.42	0.69	0.51	0.47	0.70
Date	0.50	0.61	0.73	0.60	0.69	0.76
Region	0.99	1.00	0.99	0.98	1.00	0.98
Restriction	0.36	0.43	0.74	0.74	0.58	0.69
Type + Date	0.44	0.53	0.70	0.51	0.59	0.72
Type + Value + Date	0.31	0.34	0.62	0.35	0.36	0.59
Type + Value + Date + Region	0.30	0.33	0.62	0.35	0.36	0.59
Type + Value + Date + Region + Restriction	0.26	0.29	0.61	0.34	0.35	0.59

### Future directions

We note that the recall of NPI can be improved by using Wikipedia articles in the regional languages. This could be implemented by either employing a multilingual NPI extraction model or by machine-translating the original regional language article and then applying the current NPI extraction model trained on the English language Wikipedia articles. Expanding beyond Wikipedia to other data sources would also improve the coverage. Accuracy of the pipeline could be improved by using end-to-end entity linking techniques for entity normalization and state-of-the-art methods for better temporal alignment. Aligning the taxonomy of WNTRAC with other NPI datasets will improve the interoperability of studies. A coarse-grained mapping of WNTRAC taxonomy to OxCGRT and CoronaNet datasets is available in the Supplementary Information file. We recognize that the convergence of taxonomies into a universal coding system based on say, public health and social measures PHSM^18^ dataset from World Health Organization (WHO), will help integrate several international NPI datasets.

## Usage Notes

The primary objectives of creating the WNTRAC dataset are to understand what types of NPIs are being implemented worldwide, to describe when they are imposed and lifted, and to facilitate analysis of their efficacy. Specifically, the dataset supports a variety of studies, such as correlation analysis to understand the associations between NPIs and outcomes, causal inference to draw conclusions about causal relationships between NPIs and specific outcomes, and impact analysis to understand the effects of NPIs on different socio-economic factors. In addition, this dataset can be used with other tools to support “What-if” scenario analysis and COVID-19 NPI planning. Such studies and analyses are critical for decision-makers to determine which set of NPIs to impose and lift at what points in time to help control the spread of the disease and minimize negative impact on society.

**Fig. 9:**
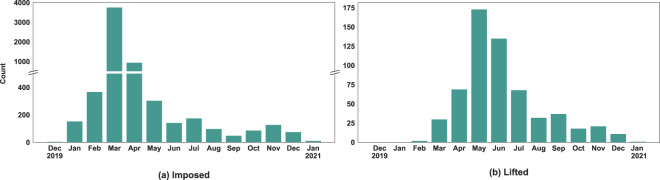
Number of imposed and lifted NPIs per month.

There are a number of questions, ranging in complexity, that this dataset may be used to answer. For example, consider the question: How many NPIs were imposed and lifted globally over a specific time frame? Fig. 9 illustrates the total number of NPIs imposed and lifted in all geographies per month. As expected, the majority of NPIs were imposed early in the pandemic in March 2020, and lifted mainly in April and May 2020. This figure also reveals the imbalance between imposed and lifted NPIs that exist in the data. For example, while more than 3,000 NPIs were imposed during March, less than 500 were lifted between April and September. This imbalance may be because of many factors, such as how and when lifting of NPIs is announced over time and missing or undocumented data. Such factors should be considered when performing analysis using this dataset.

**Fig. 10:**
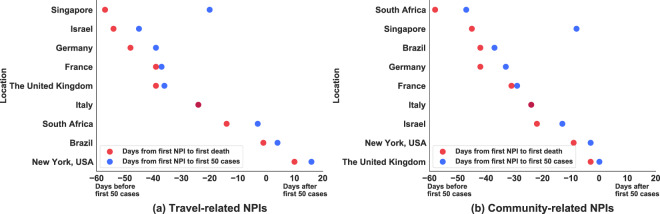
Elapsed time (in days) between the introduction of NPIs and recording of first death (red) or 50 cases (blue) in countries that implemented travel-related vs community-related NPIs first. A negative value implies that the first travel or community NPI was imposed prior to the first death (or 50 confirmed cases) while a positive value means that the NPI was imposed after such an event. For example, Israel, Germany and France imposed a travel related NPI over a month before reaching 50 confirmed cases or first death, while in USA-NY such NPI was imposed only 10 days after the first death. In most countries the first NPI was imposed before reaching 50 confirmed cases and before the first death occurred.

A second example for use of the dataset is to explore *which NPIs were imposed by different countries early in the pandemic, to contain the spread of COVID-19*. One approach is to break the set of NPIs into two sets: travel-related and community-related. Travel-related NPIs include *domestic flight restrictions*, *international flight restrictions*, *freedom of movement (nationality dependent)*, and *introduction of travel quarantine policies*. The community-related NPIs include *entertainment/cultural sector closure*, *confinement*, *school closure*, *mass gatherings*, *mask-wearing*, *public services closure*, *public transportation*, *work restrictions*, and *state of emergency*. Figure 10 depicts the number of days between the implementation of travel-related NPI vs community-related NPI and two outcomes of interest: (i) the recording of at least 50 cases (shown with blue points), and (ii) the first reported death due to COVID-19 (shown with red points). In the figure, days less than zero denote the number of days prior to the outcome of interest. The number of days greater than 0 denote the amount of time that passed after the specific outcome. We selected 9 regions, each of which had at least one travel-related NPI among the first set of NPIs imposed in the country, by combining the WNTRAC dataset with the COVID-19 outcomes dataset from the WHO (https://covid19.who.int/) and the CDC (https://www.cdc.gov/coronavirus/2019-ncov/cases-updates/cases-in-us.html). For each selected region, the blue points illustrate the number of days before the first 50 reported cases. For example, according to our dataset, Brazil imposed at least one community-related NPI 40 days prior to their first 50 cases being reported. The red points show the number of days before (or after) the first death occurred. From the graph, it can be observed that Singapore first imposed a travel-related NPI more than 50 days before their first death, showing an earlier response than Brazil and New York State. In New York State the first travel-related NPIs were imposed about 10 days after the first reported COVID-19 death. It can also be noted from Fig. 10 that at least one community-related NPI was imposed for each of the selected regions prior to their first recorded death and the first 50 cases.

As a third example, we demonstrate how the WNTRAC dataset can be used to define a composite index, called the WNTRAC NPI Index, which combines the stringency level of the NPI imposed for a region with a notion of “compliance” as defined by how well the population complies to the NPIs on a given day. While a stringency index, *SI*, has been defined by OxCGRT^12^, it does not incorporate a notion of behavior, specifically public compliance to the NPIs. Compliance to NPIs, in addition to stringency, is an important indicator for understanding the transmission of COVID-19. To develop the WNTRAC NPI Index, we propose a mapping of the WNTRAC data types to the data types used to compute *SI*, and a formula for representing compliance to the NPIs imposed using observed mobility data such as the Google’s community mobility reports (https://www.google.com/covid19/mobility) and the Apple’s COVID-19 mobility trend reports (https://covid19.apple.com/mobility) as a proxy. The two measures are combined to better reflect on-the-ground NPI compliance for a region. For example, a country may have a high stringency index, but the public may not have changed their behavior and remain very mobile, despite the stringent NPIs imposed. The aim of the index is twofold: (i) to provide a summary measure of stringency and compliance for a region at a given point and time, and (ii) to incorporate into COVID-19 epidemiological models with the goal of improving model predictions (this can be used downstream for NPIs planning and decision-making).

The WNTRAC NPI Index, *v*(*t*) ∈ [0, 1], is presented in Eq. 1 where the first term refers to the stringency index, *SI*, and the second term represents *Compliance-Score*, *CS*. Each term has a pre-specified weight denoted by *ω*_0_ and ω_1_ respectively, where *ω*_0_, ω_1_ > 0 and *ω*_0_ + ω_1_ = 1. For a given location and time, a value of 1 represents a high stringency index where the public was compliant, and a 0 represents a low stringency index with the public compliance at near pre-pandemic levels. Note, COVID-19 outcomes such as the number of reported cases are not included in the index, and the index does not represent a causal relationship between NPI and outcomes. Instead, the WNTRAC NPI Index serves as a way to measure or approximate the public interaction for a specific region on a given day, considering the NPIs that have been imposed and public mobility. The index may support analysis and modelling efforts to understand the spread of the disease based on the public’s behavior.

1 $$\nu (t,l)={\omega }_{0}SI(t,l)+{\omega }_{1}CS(SI,t,l)$$

2 $$CS(SI,t,l)=\left\{\begin{array}{ll}\frac{{e}^{c(SI,t,l)}-{e}^{-1}}{1-{e}^{-1}}, & -{\rm{1}} < c{\rm{(}}SI,t,l{\rm{)}} < {\rm{0}}\\ 1, & {\rm{otherwise}}\end{array}\right.$$

3 $$c(SI,t,l)=\frac{{m}_{ant}(SI,t,l)-{m}_{obs}(t,l)}{\left|{m}_{ant}(SI,t,l)\right|}.$$

We describe our intuition for the *Compliance-Score* by first defining *compliance*, *c*(*SI, t, l*) in Eq. 3. Provided with a baseline of “pre-pandemic” mobility levels equal to 0 (as in the Google mobility data), we let *m*_*obs*_ be the “observed mobility” which represents the percent change in mobility for a population from the baseline. In general, *m*_*obs*_ < 0. This is shown in the Google mobility data for the US, where values of *m*_*obs*_ > 0 are rare. We assume a linearly decreasing relationship between stringency and observed mobility. A lower *SI* yields a lower change in the mobility and a high *SI* yields a much larger change in mobility from the baseline (i.e., a value much less than the baseline of 0). Given this relationship, we define a notion of “anticipated mobility” for a given stringency index, *m*_*ant*_(*SI, t, l*), to be a value generated from the decreasing linear relationship with added Gaussian noise. This relationship can be learned using historical stringency levels and observed mobility for a specific location. Compliance *c*(*SI, t, l*) is then a function of the anticipated mobility, *m*_*ant*_ derived from a given stringency index, and the observed mobility, *m*_*obs*_ for the location. As a result, if the population has an observed mobility equal to the anticipated mobility, then intuitively, their change in mobility is “as expected or anticipated”, thus *c*(*SI, t, l*) = 0. If the observed mobility for the population is lower than anticipated, then *c*(*SI, t, l*) > 0, and the public’s compliance to the NPIs is “better” than anticipated. For example, if *m*_*ant*_ = −35% and *m*_*obs*_ = −50% then the population is moving much less than anticipated.

**Fig. 11:**
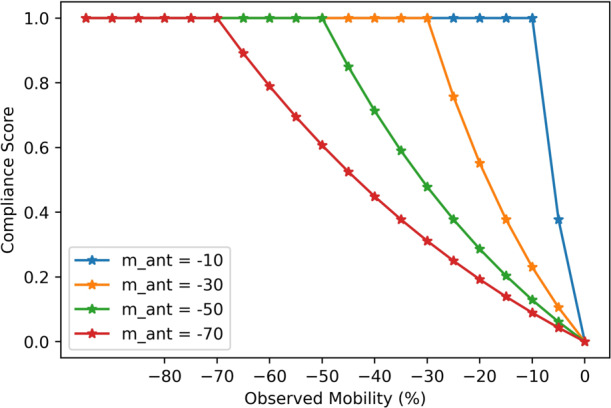
Example of the Compliance-Score for different levels of anticipated mobility, where a lower anticipated mobility (e.g., *m*_*ant*_ = −10%) is derived from a lower stringency index, and higher anticipated mobility (e.g., *m*_*ant*_ = −70%) is derived from a higher stringency index. A Compliance Score of 0 refers to a poor level of public compliance to the NPIs imposed for a given location, and a score of 1 implies that the public is complying to the NPIs imposed given the stringency index for a location.

Given our definition of compliance, we define the Compliance-Score *CS*(*SI, t, l*) in Eq. 2, as a mapping of compliance values to [0, 1]. According to the equation, when *c*(*SI, t, l*) ≥ 0 representing that the public is behaving as expected or better, then *CS*(*SI, t, l*) = 1. Equation 2 penalizes lower compliance values, accordingly. For example, if a location has a high stringency index yielding an anticipated mobility *m*_*ant*_ = −50%, and if the observed mobility is close to pre-pandemic levels, e.g., *m*_*obs*_ = −5%, then compliance is −0.9 and the Compliance Score is 0.06, very close to 0 representing “poor” compliance. Figure 11 presents an example of Compliance-Scores for varying levels of anticipated mobility derived from the linear relationship with the stringency index of a location.

**Fig. 12:**
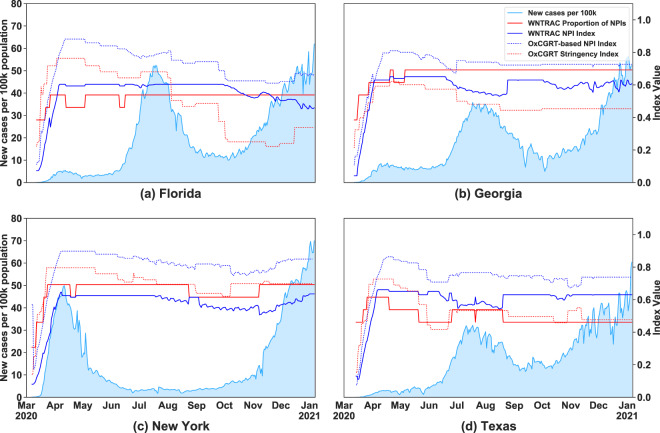
Trends in COVID-19 cases per 100,000 population since first 50 cases and the NPI-based indices in representative US states. At the beginning of the pandemic the 4 states shown have similar trends in the NPI index values. As the pandemic progressed, particularly by October 2020, there was as decrease in the NPI index values that appears to be associated with a subsequent increase in new COVID-19 cases.

Figure 12 illustrates the differences in indices using data from representative states in the United States (Florida, Georgia, New York, and Texas). In the figure, the light blue graph shows the trend for the exponentially weighted moving average of new cases per 100,000 population. The red continuous line is the proportion of the NPIs out of 13 NPI types in the WNTRAC dataset that a region has imposed at a given time. The blue continuous line is the NPI Index computed after manually mapping the WNTRAC NPI data types to the OxCGRT NPI categories. The code to generate the mappings and to calculate the indices is included in our repository at https://github.com/IBM/wntrac/blob/master/code/analysis/NPI_index.ipynb. The red dotted line is the OxCGRT *SI* while the blue dotted line is the computation of the NPI Index using the OxCGRT NPI dataset. One way of interpreting the graphs is that if the WNTRAC NPI index is higher than the OxCGRT *SI*, then it is likely that the public compliance to the imposed NPIs was better than anticipated.

Another important application of the WNTRAC dataset is to support “What-if” scenario analysis and decision-making for optimal intervention planning. An accurate and comprehensive picture of the NPIs imposed in a region is especially important to provide critical, time-sensitive decision-support to leaders and decision-makers who make-up COVID-19 task force teams. Such teams determine which NPIs to impose or lift over time and also have to examine the varying degrees of impact the NPIs might have on COVID-19 outcomes for a region. As the space of all potential combinations of NPIs can be large and complex, intervention planning tools such as the one noted by Wachira *et al*.^19^ may be used with the WNTRAC dataset for NPI planning and decision-support support at national and sub-national levels. For decision-makers, these tools enable easy navigation through the complex intervention space to generate the optimal and contextually-relevant COVID-19 NPI plans. A key requirement for such tools are epidemiological models. By incorporating NPIs into the models, such as with the proposed WNTRAC NPI Index, improved projections of the disease outcomes can be generated, yielding more accurate scenarios for decision-makers to explore.

In addition to the above examples, the WNTRAC dataset can be used to support other objectives, including estimating the relationships between NPIs and consumer’s behavior by, for example, correlating between retail data and NPIs or environmental changes such as pollution levels. Additionally, the WNTRAC dataset can be used to study actual compliance by the population. Naturally, however, not all the interventions recorded in the dataset are an accurate representation of reality as some of the interventions capture a governmental request that might not be followed by the entire population. Thus, it might be useful to integrate the WNTRAC dataset with other publicly available data sources that can provide information regarding the level of compliance with an intervention, such as mobility information as exemplified by the NPI Index discussed above. Lastly, one other interesting use case is to estimate the economic impact of NPIs, for example, by relating unemployment rates and jurisdictional debt with NPIs. Estimating the effect of NPIs on non-COVID-19 health problems, such as late cancer detection due to missed screening tests, will also be useful.

## Supplementary information

Supplementary Information File

## Data Availability

The source code for the WNTRAC automated NPI curation system, including the data processing pipeline, WNTRAC Curator tool and NPI data browser is available in a public GitHub repository at https://github.com/IBM/wntrac/tree/master/code alongside the up-to-date version of the dataset https://github.com/IBM/wntrac/tree/master/data. Please refer to the README file in repository for further instructions on using the code.
