# Supplementary material for: AI-assisted tracking of worldwide non-pharmaceutical interventions for COVID-19
Source: Sci Data. 2021 Mar 25;8:94. doi: 10.1038/s41597-021-00878-y (PMC7994304; doi:10.1038/s41597-021-00878-y)
Supplement: Supplementary file 1 — Supplementary Information File [file 41597_2021_878_MOESM1_ESM.pdf]

Contents

1 Dataset Comparison 2
2 Taxonomy Comparison 3
3 Geographical Coverage 4

1 Dataset Comparison

Dataset	Data collection method	Coverage	Date range
Complexity Science Hub Covid-19 Control Strategies List (CCCSL)	Manual - over 40 volunteers	60 countries	2019-12-31 to 2021-01-01
CoronaNet	Hybrid, over 500 scholars and research assistants	195 countries	2019-12-31 to 2021-01-10
Oxford COVID-19 Government Response Tracker (OxCGRT)	Manual - over 450 Oxford University students and staff	185 countries	2020-01-01 to 2021-01-13
ACAPS - COVID-19 Government Measures	Manual - ACAPS analysts and volunteers from University of Copenhagen and University of Lund	193 countries	2020-01-01 to 2020-12-08
Response2covid19	Manual	229 countries	2020-01-01 to 2020-08-31
HIT-COVID	Manual - over 200 volunteers from Johns Hopkins Bloomberg School of Public Health, Boston University School of Public Health	155 countries	2019-12-31 to 2021-01-11
Worldwide Non-pharmaceutical Interventions Tracker for COVID-19 (WNTRAC)	AI-assisted, human-in-the-loop incremental non-pharmaceutical intervention (NPI) curation system powered by Wikipedia. Daily incremental validation performed by 2 volunteers working 1 hour per day. Initial validation was done by 37 IBM volunteers to bootstrap the system.	261 regions including 54 US states and territories	2020-01-01 to 2021-01-06

Table 1. Comparison of prominent NPI datasets accessed as of 11th, January 2021.

2 Taxonomy Comparison

OxCGRT			CoronaNet		WNTRAC	
**Category**	**Indicator**	**NPI count**	**Policy type**	**NPI count**	**NPI type**	**NPI count**
C - containment and closure	C1 - School Closures	1025	Closure and Regulation of Schools	1449	School Closures	964
	C2 - Workplace closing	1244	Restriction and Regulation of Businesses	3210	Work Restrictions	268
			Restriction and Regulation of Government Services	1531	Public Service Closures	101
					Prison-related Policies	11
	C3 - Cancel Public Events	771			Entertainment/Cultural Sector Closures	1678
	C4 - Restrictions on gatherings	1077	Restrictions of Mass Gatherings	2075	Restrictions on Gatherings	270
	C5 - Public Transportation	613			Public Transportation	130
	C6 - Stay at Home Order	1021	Quarantine	1510	Confinement	1040
			Lockdown	1091		
			Curfew	498		
	C7 - Restrictions on Internal Movement	887	Internal Border Restriction	937	Domestic Flight Restrictions	47
	C8 - International Travel Controls	1086	External Border Restrictions	1912	International Flight Restrictions	457
					Travel Quarantine Policies	364
					Freedom of Movement (International)	884
E - economic	E1 - Income Support	572			Economic Impact	237
	E2 - Debt/contract relief for households	477				
	E3 - Fiscal Measures	1541				
	E4 - International Support	127				
H - health system	H1 - Public Information Campaigns	446	Public Awareness Measures	1816		
			Anti-Disinformation Measures	249		
	H2 - Testing Policy	697	Health Testing	1102		
	H3 - Contact tracing	518	Health Monitoring	1016	Contact Tracing	34
	H4 - Emergency investment in healthcare	522	Health Resources	3967		
	H6 - Facial coverings	682	Hygiene	796	Mask Wearing	186
	H7 - Vaccination policy	155	Vaccines	8		
M - miscellaneous	M - Miscellaneous policies		Declaration of Emergency	629	State of Emergency	280
			Other Policy Not Listed Above	1971	Other	excluded
			New Task Force, Bureau or Administrative Configuration	1083		
			Social Distancing	1330		

Table 2. A coarse-grained mapping of taxonomy between Oxford COVID-19 Government Response Tracker (OxCGRT), WNTRAC and CoronaNet datasets, organized by the top level OxCGRT categories. Counts of NPI based on the version of datasets accessed on 11th January, 2021. Counts for OxCGRT were derived from the time-series by considering change in stringency level for particular NPI type as an NPI event. Counts for CoronaNet are based on the unique policy id field. Counts for WNTRAC are based on the unique 5-tuple that defines an NPI as discussed in the Methods section. The Jupyter notebook available at `https://github.com/IBM/wntrac/blob/master/code/analysis/dataset_comparison.ipynb` contains the code for this comparison.

3 Geographical Coverage

Region	Code
Abkhazia	GEO-AB
Afghanistan	AFG
Albania	ALB
Algeria	DZA
Andorra	AND
Angola	AGO
Anguilla	AIA
Antarctica	ATA
Antigua and Barbuda	ATG
Argentina	ARG
Armenia	ARM
Australia	AUS
Austria	AUT
Azerbaijan	AZE
Bahamas	BHS
Bahrain	BHR
Bangladesh	BGD
Barbados	BRB
Belarus	BLR
Belgium	BEL
Belize	BLZ
Benin	BEN
Bhutan	BTN
Bolivia	BOL
Bosnia and Herzegovina	BIH
Botswana	BWA
Brazil	BRA
Brunei	BRN
Bulgaria	BGR
Burkina Faso	BFA
Burundi	BDI
Cambodia	KHM
Cameroon	CMR
Canada	CAN
Cape Verde	CPV
Central African Republic	CAF
Chad	TCD
Chile	CHL
China	CHN-TB
Colombia	COL
Comoros	COM
Costa Rica	CRI
Croatia	HRV
Cuba	CUB
Cyprus	CYP
Czech Republic	CZE
Democratic Republic of Congo	COD
Denmark	DNK
Djibouti	DJI
Dominica	DMA
Dominican Republic	DOM
Ecuador	ECU
Egypt	EGY
El Salvador	SLV
Equatorial Guinea	GNQ
Eritrea	ERI
Estonia	EST
Eswatini	SWZ
Ethiopia	ETH
Faroe Islands	DEN-FI
Fiji	FJI
Finland	FIN
France	FRA
French Guiana	GUF
French Polynesia	PYF
French Saint Martin	MAF
Gabon	GAB
Gambia	GMB
Georgia (country)	GEO
Germany	DEU
Ghana	GHA
Greece	GRC
Greenland	DEN-GR
Grenada	GRD
Guadeloupe	FRA-GU
Guatemala	GTM
Guinea	GIN
Guinea-Bissau	GNB
Guyana	GUY
Haiti	HTI
Honduras	HND
Hong Kong	CHN-HK
Hungary	HUN
Iceland	ISL
India	IND
Indonesia	IDN
Iran	IRN
Iraq	IRQ
Israel	ISR
Italy	ITA
Ivory Coast	CIV
Jamaica	JAM
Japan	JPN
Jordan	JOR
Kazakhstan	KAZ
Kenya	KEN
Kosovo	KOS
Kuwait	KWT
Kyrgyzstan	KGZ
Laos	LAO
Latvia	LVA
Lebanon	LBN
Lesotho	LSO
Liberia	LBR
Libya	LBY
Liechtenstein	LIE
Lithuania	LTU
Luhansk People's Republic	UKR-09
Luxembourg	LUX
Madagascar	MDG
Mainland China	CHN
Malawi	MWI
Malaysia	MYS
Maldives	MDV
Mali	MLI
Malta	MLT
Martinique	FRA-MA
Mauritania	MRT
Mauritius	MUS
Mayotte	FRA-MA
Mexico	MEX
Moldova	MDA
Monaco	MCO
Mongolia	MNG
Montenegro	MNE
Morocco	MAR
Mozambique	MOZ
Myanmar	MMR
Namibia	NAM
Nepal	NPL
Netherlands	NLD
New Caledonia	FRA-NC
New Zealand	NZL
Nicaragua	NIC
Niger	NER
Nigeria	NGA
North Korea	PRK
North Macedonia	MKD
Northern Cyprus	CYP
Norway	NOR
Oman	OMN
Pakistan	PAK
Panama	PAN
Papua New Guinea	PNG
Paraguay	PRY
Peru	PER
Philippines	PHL
Poland	POL
Portugal	PRT
Qatar	QAT
Republic of Artsakh	ARM
Republic of Congo	COG
Republic of Ireland	IRL
Romania	ROU
Russia	RUS
Rwanda	RWA
Saint Helena	SHN
Saint Kitts and Nevis	KNA
Saint Lucia	LCA
Saint Pierre and Miquelon	SPM
Saint Vincent and Grenadines	VCT
San Marino	SMR
Saudi Arabia	SAU
Senegal	SEN
Serbia	SRB
Seychelles	SYC
Sierra Leone	SLE
Singapore	SGP
Slovakia	SVK
Slovenia	SVN
Somalia	SOM
Somalia	SOM
South Africa	ZAF
South Korea	KOR
South Sudan	SSD
Spain	ESP
Sri Lanka	LKA
State of Palestine	PSE
Sudan	SDN
Suriname	SUR
Sweden	SWE
Switzerland	CHE
Syria	SYR
Taiwan	TWN
Tajikistan	TJK
Tanzania	TZA
Thailand	THA
Timor-Leste	TLS
Togo	TGO
Trinidad and Tobago	TTO
Tunisia	TUN
Turkey	TUR
Turkmenistan	TKM
Uganda	UGA
United Arab Emirates	ARE
United Kingdom	GBR
United States	USA
Uruguay	URY
Uzbekistan	UZB
Vatican City	VAT
Venezuela	VEN
Vietnam	VNM
Western Sahara	ESH
Yemen	YEM
Zambia	ZMB
Zimbabwe	ZWE

Table 3. List of regions currently supported by the WNTRAC dataset.

Region	Code
Alabama	USA-AL
Alaska	USA-AK
Arizona	USA-AZ
Arkansas	USA-AR
California	USA-CA
Colorado	USA-CO
Connecticut	USA-CT
Delaware	USA-DE
Florida	USA-FL
Georgia (U.S. state)	USA-GA
Guam	USA-GU
Guantanamo Bay Naval Base	USA-Guantanamo_Bay_Naval_Base
Hawaii	USA-HI
Idaho	USA-ID
Illinois	USA-IL
Indiana	USA-IN
Iowa	USA-IA
Kansas	USA-KS
Kentucky	USA-KY
Louisiana	USA-LA
Maine	USA-ME
Maryland	USA-MD
Massachusetts	USA-MA
Michigan	USA-MI
Minnesota	USA-MN
Mississippi	USA-MS
Missouri	USA-MO
Montana	USA-MT
Nevada	USA-NV
New Hampshire	USA-NH
New Jersey	USA-NJ
New Mexico	USA-NM
New York (state)	USA-NY
North Carolina	USA-NC
North Dakota	USA-ND
Northern Mariana Islands	USA-Northern_Mariana_Islands
Ohio	USA-OH
Oklahoma	USA-OK
Oregon	USA-OR
Pennsylvania	USA-PA
Puerto Rico	USA-PR
Rhode Island	USA-RI
South Carolina	USA-SC
South Dakota	USA-SD
Tennessee	USA-TN
Texas	USA-TX
United States Virgin Islands	USA-Virgin_Islands
Utah	USA-UT
Vermont	USA-VT
Virginia	USA-VA
Washington (state)	USA-WA
Washington D.C.	USA-DC
West Virginia	USA-WV
Wisconsin	USA-WI
Wyoming	USA-WY

Table 4. List of US states and territories currently supported by the WNTRAC dataset.
